# Neurophysiological Correlates of Near-Wins in Gambling: A Systematic Literature Review

**DOI:** 10.1007/s10899-024-10327-1

**Published:** 2024-08-05

**Authors:** Artemisa Rocha Dores, Miguel Peixoto, Carina Fernandes, Andreia Geraldo, Mark D. Griffiths, Fernando Barbosa

**Affiliations:** 1 Laboratório de Reabilitação Psicossocial - Centro de Investigação em Reabilitação (LabRP-CIR), Escola Superior de Saúde, Polytechnic Institute of Porto, Rua Dr. António Bernardino de Almeida, 400, Porto, 4200-072 Portugal; 2 Laboratory of Neuropsychophysiology, Faculty of Psychology and Education Sciences, University of Porto, Rua Alfredo Allen, Porto, 4200-135 Portugal; 3 Faculty of Human and Social Sciences, University Fernando Pessoa, Porto, Portugal; 4 International Gaming Research Unit, Psychology Department, Nottingham Trent University, 50 Shakespeare Street, Nottingham, UK

**Keywords:** Gambling, Near miss, Near Win, Event-related potentials (ERP), P300, Feedback-related negativity (FRN)

## Abstract

Identification of specific patterns of brain activity related to problem gambling may provide a deeper understanding of its underlying mechanisms, highlighting the importance of neurophysiological studies to better understand development and persistence of gambling behavior. The patterns of cognitive functioning have been investigated through electroencephalography (EEG) studies based on the near-win/near-miss (NW) effect. The main goal of the present study was to evaluate the neurophysiological basis of NWs and their modulation by gambling problems through a systematic review of event-related potentials (ERP) studies elicited by feedback events. The review followed the recommendations of the Preferred Reporting Items for Systematic Reviews and Meta-Analysis Protocols (PRISMA). A total of 15 studies were included, 12 comprising non-problem gamblers (NPGs) and three comparing problem gamblers (PGs) with matched controls. For the P300 component, the win outcome elicited a larger amplitude than the other outcomes (NW and loss), followed by the NW outcome, which elicited a larger amplitude than loss in some studies. For feedback-related negativity (FRN), the loss outcome evoked a more negative amplitude in several studies, despite eliciting a similar amplitude to NW outcomes in others. For PGs, the NW outcome evoked a higher amplitude of P300 than loss, while NPGs showed a similar amplitude to both outcomes. The present review gathered information from different sources and provides a consistent view of the different studies. However, studies lack systematic and robust methodologies, leading to inconsistent results and making it difficult to reach any definitive conclusions.

## Introduction

Gambling involves the staking of money or something of value in an event with an uncertain outcome, in the hope of winning additional rewards (Williams et al., 2017). This act of risk-taking can lead to stimulation and is reinforced by the emotional experience that follows (National Research Council, 1999).

In the contemporary landscape, the proliferation of online platforms has led to unprecedented accessibility to various forms of entertainment and activities, including gambling. However, alongside this accessibility comes the rise of gambling disorder (American Psychiatric Association [APA], 2013; World Health Organization [WHO], 2023), with detrimental consequences for individuals and society (Fineberg et al., 2018, 2022) that are becoming a matter of public health (Ukhova et al., 2024). In the DSM-5 (APA, 2013), gambling disorder was included in the “Substance-Related and Addictive Disorders” section, under the “Non-Substance Related Disorders” sub-section. It is currently considered a behavioral addiction (APA, 2013) or a disorder due to addictive behaviors (WHO, 2023), which is characterized as a behavior that reinforces itself, and is associated with high impulsivity (Robbins & Clark, 2015). Gambling disorder is characterized by increasing the amount of money gambled to feel excitement, failing to reduce gambling expenditure, disruption of daily life and finances, lying to others about involvement with gambling, and repeated gambling activity to get even (i.e., chasing losses) (APA, 2013).

According to a meta-analysis by Bijker et al. (2022), it has been estimated that gambling disorder impacts between 0.6% and 2% of the global population. As stated by the Sustainable Development Goals (SDGs) outlined in the 2030 Agenda, it is crucial to promote ‘good health and well-being’ (SDG 3) of the citizens (United Nations, 2015). Therefore, more and better research in the area can help to prevent or minimize the mental health impact of the disorder and its public health implications, particularly by promoting awareness about gambling issues.

As previously mentioned, gambling disorder is recognized as a mental health condition with significant public health implications, leading to adverse consequences for individuals and communities, and affecting overall well-being. However, behavioral addictions, such as gambling disorder, have received less attention compared to substance addictions, despite similarities in psychophysiology, clinical expression, brain origin, comorbidities, and treatment (Bowden-Jones, 2017). Understanding the factors contributing to the transition from recreational to excessive and pathological gambling, particularly among vulnerable populations, is essential. While the etiology of gambling disorder remains inconclusive, individual, structural, and situational factors are known to play significant roles.

Among the individual factors, specific traits of personality can be a major risk factor, having a higher predictive value than neuropsychological variables for problem gambling (Forbush et al., 2008), with high impulsivity (Mestre-Bach et al., 2020), high neuroticism, low agreeableness, low consciousness (Dudfield et al., 2022; Whiting et al., 2019), and low openness (Dudfield et al., 2022) being significant predictors of problem gambling. In terms of comorbidities, results show that depression (Barrault & Varescon, 2013; Hing et al., 2016; Nigro et al., 2019; Schluter et al., 2019) and anxiety are two predictors of problem gambling (Barrault & Varescon, 2013; Hing et al., 2016).

Other important risk factors are the structural characteristics of the most addictive games. Specific types of gambling (e.g., electronic gambling machines [EGMs]) maximize engagement, using tactics such as jackpots (Rockloff & Hing, 2013), free spins (Landon et al., 2018), generating near-win/near-miss (NW) scenarios (Rockloff & Hing, 2013), using multiple paylines (Landon et al., 2018; Murch & Clark, 2019), the use of losses disguised as wins (i.e., when the player wins less money than the amount gambled) (Barton et al., 2017), and audio-visual effects that can be used to reinforce losses disguised as wins (Barton et al., 2017). Engagement with EGMs can lead to high levels of arousal, higher expenditure, and longer play sessions among problem gamblers (PGs) compared to non-problem gamblers (NPGs) (Brown et al., 2004; Hing et al., 2016; Hing & Russell, 2020).

One of the key structural characteristics of EGMs is the NW, an event that despite being a loss, is perceived to be close to a win which can reinforce gambling behavior (Parke & Griffiths, 2004). NWs can activate brain circuits that can motivate further gambling behavior (Clark et al., 2009). Facial electromyography focused on zygomatic activity also shows that NWs can lead to a similar response pattern as a win (Wu et al., 2015). Furthermore, NWs can contribute towards the development and maintenance of dysfunctional cognitions (Clark, 2014; Clark et al., 2013), characterized by irrational or biased thoughts towards gambling (Goodie & Fortune, 2013; Johansson et al., 2009; Mathieu et al., 2018; Raylu & Oei, 2002; Yakovenko et al., 2016).

Dysfunctional cognitions are especially problematic for individuals who prefer skill games (e.g., card games) (Myrseth et al., 2010; Toneatto et al., 1997). These cognitions are related to the belief that it is possible to influence the game’s outcome or that the outcome can be predicted, which is referred to as the illusion of control (Goodie & Fortune, 2013; Johansson et al., 2009; Raylu & Oei, 2002). Besides the risk posed, dysfunctional cognitions can also lead to a higher need of immediate rewards, to more impulsive gambling behavior (MacLaren et al., 2015; Michalczuk et al., 2011), and to increased gambling motivation (Mathieu et al., 2018).

Additionally, gambling is associated with several cognitive dysfunctions. For example, attentional bias for gambling stimuli is a cognitive factor present among PGs, and that correlates with gambling frequency, beliefs, and attitudes (Ciccarelli et al., 2022; Grant & Bowling, 2015). It has also been shown that the correlation between severity and attentional bias is moderated by craving and impulsivity (Ciccarelli et al., 2022; Kim et al., 2022), and both aspects could stem from the gambler’s necessity to relieve negative emotions (Ciccarelli et al., 2022). Moreover, studies have shown that memory bias towards gambling-related behaviors is associated with problem gambling (Russell et al., 2019; Stiles et al., 2017). Prospective memory worsens with gambling severity, which in turn leads to increased gambling severity (Nigro et al., 2019).

When it comes to executive functions, response inhibition is affected among PGs (Forbush et al., 2008; Odlaug et al., 2011), influencing delayed discounting (i.e., the way rewards are depreciated based on the time they take to be obtained). More specifically, these individuals tend to choose immediate rewards over larger ones that would take longer to achieve (Da Matta et al., 2012; Kräplin et al., 2014; Mestre-Bach et al., 2020; Ochoa et al., 2013). The differences in executive functions between PGs and NPGs vary across studies, with some reporting no differences between PGs and NPGs (Kapsomenakis et al., 2018; Marazziti et al., 2008), only identifying PGs as more impulsive (Kapsomenakis et al., 2018), or as having higher inflexibility (Marazziti et al., 2008).

Related to the aforementioned factors, decision-making has been shown to be impaired among PGs (Achab et al., 2014; Kräplin et al., 2014; Mestre-Bach et al., 2020; Ochoa et al., 2013), especially under scenarios of uncertainty or risk, with a lack of explicit knowledge about the game (Achab et al., 2014; Ochoa et al., 2013), or in cases of comorbid depression (Rimal et al., 2022). Higher activation of reward pathways among gamblers for wins and NWs can also lead to impairment in decision-making (Achab et al., 2014; Ochoa et al., 2013). Hyperactivation of reward pathways is also seen in high-risk scenarios, in contrast to hypoactivation in low-risk scenarios (Achab et al., 2014). When it comes to rewards, PGs tend towards risk-seeking choices (tendency to value a likely larger gain more than a certain smaller gain (Shead & Hodgins, 2009). They go for rewards that have a lower chance of being obtained, showing a lower degree of probability discounting (Wiehler & Peters, 2015).

One of the methods used to gather information on how the brain operates while gambling or in response to gambling stimuli is electroencephalography (EEG) and the event-related potentials (ERPs) that can be computed from the former. ERPs are small registered voltage oscillations, time-locked to the presentation of a stimulus (Donchin & Coles, 1988; Huang et al., 2015; Kropotov, 2016). ERP activity is usually classified based on the latency and amplitude of the constituent waves or components. The P300 is one of the most studied ERP components. P300 is a centroparietal positive wave that peaks approximately at 300ms post-event, and in processing changes in the environment, i.e., to show greater amplitude in response to stimuli with motivational salience or to novel/unexpected stimuli (Donchin & Coles, 1988; Huang et al., 2015; Rugg & Coles, 1995). When elicited by monetary wins/losses, P300 shows a bigger amplitude for these outcomes compared to trials with no monetary rewards (Broyd et al., 2012).

On the other hand, feedback-related negativity (FRN) is a frontocentral negative deflection that occurs between 200-300ms and is the basis of reinforcement learning (Talmi et al., 2013). Initially, FRN was thought to be a reaction exclusive to error detection, which would then be used to learn and improve the behavior, being tied to inhibitory behavior (Falkenstein et al., 2000; Holroyd & Coles, 2002; Miltner et al., 1997). However, a more recent perspective shows that FRN activity is related to expectancy/outcome prediction, where a bigger break of expectations leads to a more negative amplitude (Alexander & Brown, 2011; Ferdinand et al., 2012; Hauser et al., 2014; Oliveira et al., 2007; Talmi et al., 2013; Walsh & Anderson, 2012). FRN also reflects reward valence, where more negative outcomes produce more negative FRN amplitudes (Broyd et al., 2012; Kóbor et al., 2015; Walsh & Anderson, 2012).

Despite the relevance of neurophysiological studies, there is a lack of consistency in their results, and gaps in their findings to understand the acquisition, development, and maintenance of excessive and problem gambling. For example, although various neurophysiological studies have explored these behaviors and their neuronal correlates or predictors, phenomena such as NWs remains unclear, requiring further investigation, especially considering that they can be intentionally introduced by game designers (Palmer, Ferrari, & Clark., 2024). Neurophysiological techniques could provide further insight into gambling behavior, but few studies employ them. Moreover, the methodologies used are heterogeneous. Studies in decision-making also lack more extensive use of brain measures to better understand the hyperactivation of reward pathways to wins, NWs, and high-risk scenarios, among PGs (Achab et al., 2014; Ochoa et al., 2013). To the best of the authors’ knowledge, this is the first systematic review to evaluate studies that have used ERPs to study gambling behavior. By doing so, it is expected to better understand what neuronal mechanisms are involved in the development and maintenance of a gambling disorder, while also providing a more consistent view of the literature to inform future research. The following research questions were formulated:

### Research Question 1 (RQ1):

What are the specific neuropsychophysiological mechanisms underlying excessive and problematic gambling behaviors?

### Research Question 2 (RQ2):

What is the role of NWs in reinforcing gambling behavior?

### Research Question 3 (RQ3):

How do P300 amplitudes vary in response to gambling paradigms and various gambling outcomes?

### Research Question 4 (RQ4):

How do FRN amplitudes vary in response to gambling paradigms and various gambling outcomes?

### Research Question 5 (RQ5):

How do P300 and FRN amplitudes vary between PGs and NPGs?

### Research Question 6 (RQ6):

What role do behavioral studies play in the research of gambling behavior?

## Method

The present review followed the recommendations of the Preferred Reporting Items for Systematic Reviews and Meta-Analysis (PRISMA; Page et al., 2021) and Cochrane collaboration guidelines (Higgins & Green, 2019). The PRISMA statement provides a widely recognized guideline designed to enhance the transparency and quality of systematic reviews and meta-analyses. Developed by a group of experts, PRISMA provides a set of essential items to include in these types of studies, ensuring a clear and comprehensive presentation of methods and results. The adoption of PRISMA helps minimize bias, facilitate study replication, and improve the reliability of scientific evidence (Moher et al., 2009).

### Search Strategy

A systematic search was conducted in 2023 to find studies that had analyzed EEG activity in gambling tasks. The search was conducted using the *b-on* search engine, using the filter “AB Abstract”, and an automatic filter for duplicate studies. The search was carried out using the following databases: *Academic Search Complete, Scopus*, and *ScienceDirect.* Furthermore, references of the selected studies were reviewed to identify other relevant studies. To prevent publication and source selection bias, an additional hand search was performed. The search string used was the following: (near-miss OR “near miss” OR “near win” OR “near-win”) AND (electroencephalog* OR evoked potenti* OR event-related potenti* OR ERP OR EEG OR band OR oscillatory OR P300 OR FRN) AND Gam*) NOT Animal*.

### Study Selection

The following inclusion criteria were used to identify relevant studies for review: (a) had participants who were PGs and/or NPGs; (b) were original empirical studies published in English peer-reviewed journals in the last 20 years; (c) used EEG and ERP techniques; and (d) specifically analyzed P300 and FRN components elicited by an economic decision-making task. The exclusion criteria were: (a) not using a gambling related task; (b) studies with participants under 18 years of age; (c) studies with participants with major psychological comorbidities; and (d) reviews, meta-analysis, case studies, and conference presentations. After the elimination of duplicates, a full-text analysis was performed by two independent reviewers, in accordance with Higgins and Green (2019). A third reviewer was involved to resolve any disagreements.

### Procedure

Across the studies reviewed, different terms were used to refer to the same outcomes. For example, some studies used the term ‘gain’, while others used the term ‘win’, and authors varied between ‘near miss’ and ‘near win’ to refer to the same outcome. To standardize the terminology and improve clarity, the outcomes were labeled as follows: big win (BW); small win (SW); win; narrow win (NaW); near win (NW); big loss (BL); small loss (SL); and loss.

Considering that NWs can be intentionally incorporated into games of chance, possibly promoting problem gambling, the present review tackled the challenges associated with the recent reconceptualization of gambling disorder and with the methodological problems that might be leading to inconsistent findings in the literature.

## Results

**Fig. 1:**
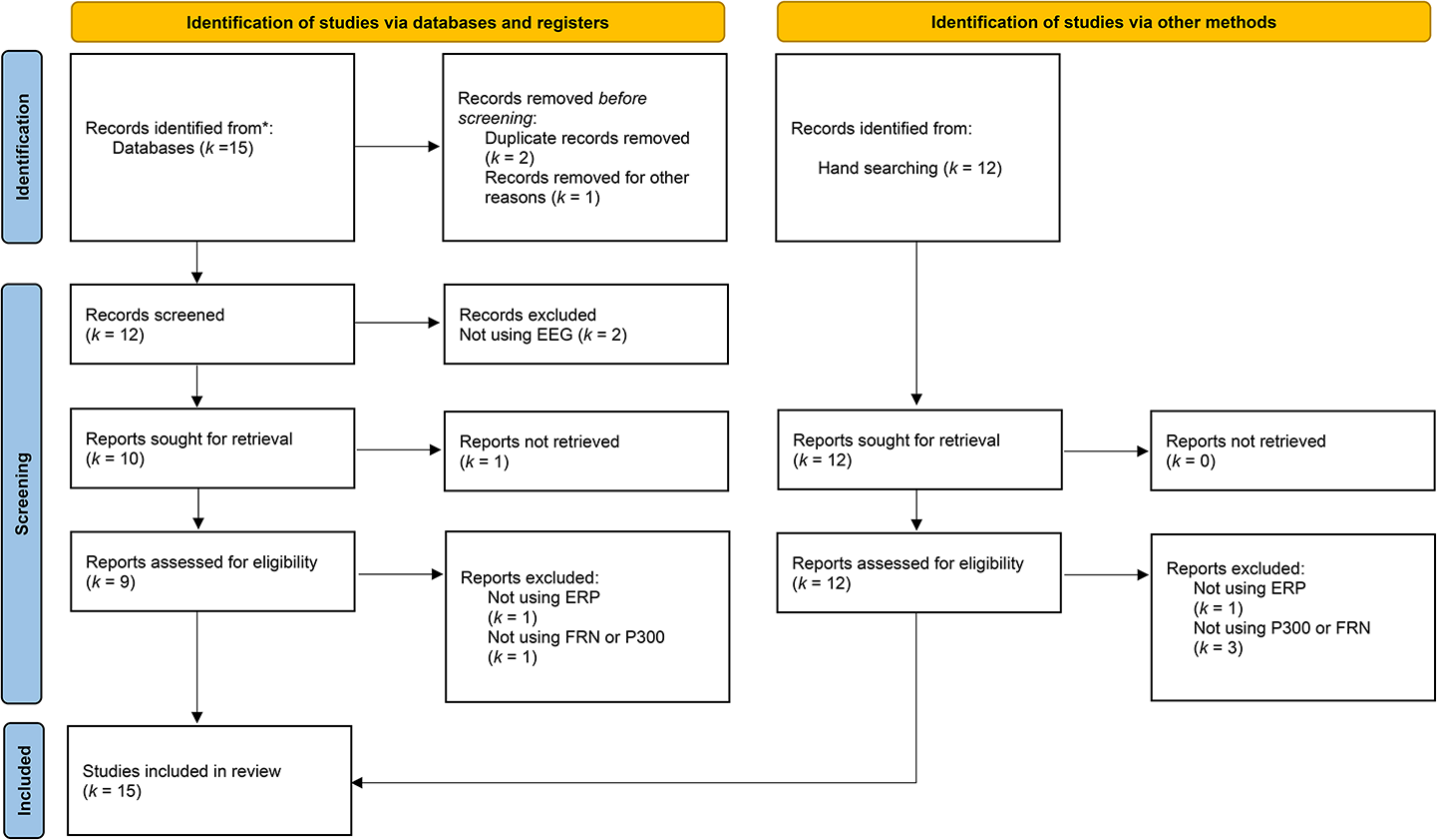
Flow diagram of literature search. (adapted from Page et al., 2021)

A total of 15 studies were identified following the systematic search of the databases. After the exclusion of duplicate papers (*k* = 2) and studies with non-related topics (*k* = 1), the title and abstract of 12 studies were screened by the reviewers. After screening, two studies were excluded due to not using EEG. Ten studies remained for a full-text analysis. When preparing for the full-text analysis, one study was excluded for not having the full-text available. Out of the nine studies submitted to a full-text analysis, there was one exclusion for not using ERP (*k* = 1), and one for not assessing FRN or P300 (*k* = 1). Therefore, seven studies were selected using a database search. An additional 12 studies were identified by manual search. After screening, four were excluded due to either not using ERP (*k* = 1) or not assessing P300 or FRN (*k* = 3). Consequently, eight studies were selected through manual search. In total, 15 studies fulfilled the inclusion criteria for further evaluation and review. The systematic review process is presented in Fig. 1.

The 15 studies were published between 2006 and 2018, with a total of 443 participants (*M* = 29.53; *SD* = 13.93; *Min* = 17; *Max* = 60), of which 182 were female (41.08%), and 243 were male (54.85%), with one study not reporting participants’ gender. Three studies compared PGs to NPGs (Kreussel et al., 2013; Lole et al., 2015; Ulrich & Hewig, 2018), and the other 12 comprised NPGs (Alicart et al., 2015; Dores et al., 2020; Hajcak et al., 2006; Hewig et al., 2007; Lole et al., 2013; Luo et al., 2011; Peterburs et al., 2013; Qi et al., 2011; Ulrich & Hewig, 2014; Wu & Zhou, 2009; Xia et al., 2018; Zhou et al., 2010), with a total of 57 PGs (12.87%) and 386 NPGs (87.13%).

Thirteen studies were conducted in five countries. Additionally, two studies were conducted simultaneously in more than one country. The characteristics of the included studies are presented in Table 1.

**Table 1:** Sociodemographic characteristics

Authors	Country	Type of Participants (*n*)	Gender (*n*)	Age M (SD) Min-Max
Hajcak et al., 2006*	Canada; USA	**Experiment 1: NPG** (16) **Experiment 2: NPG** (17)	**Experiment 1**: **M** (6) **F** (10) **Experiment 2: M** (5) **F** (12)	-----------
Hewig et al., 2007	Germany, Netherlands and Canada	**NPG** (18)	**M** (2) **F** (16)	**NG**: 20.89 (2.4) 18–26
Wu & Zhou, 2009	China	**NPG** (19)	**M** (13) **F** (6)	**NG**: 22–26
Zhou et al., 2010	China	**NPG** (18)	-----------	-----------
Luo et al., 2011	China	**NPG** (19)	**M** (8) **F** (11)	**NG**: 22 (1.63) 19–25
Qi et al., 2011	China	**NPG** (17)	**M** (8) **F** (9)	**NG**: 21.9 19–34
Kreussel et al., 2013	Germany	**NPG** (22) **PG** (21)	**NG**: **M** (22) **PG**: **M** (21)	**NG**: 23 (3.23) **PG**: 24 (4.66)
Lole et al., 2013	Australia	**NPG** (17)	**M** (7) **F** (10)	**NG**: 18.7 (4.7) 18–23
Peterburs et al., 2013	Germany	**NPG** (28)	**M** (15) **F** (13)	**NG**: 24.61 (4.76) 19–35
Ulrich & Hewig, 2014	Germany	**NPG** (60)	**M** (30) **F** (30)	**NG**: 25.23 (4.5) 18–39
Alicart et al., 2015	Spain	**NPG** (20)	**M** (8) **F** (12)	**NG**: 24.12 (4.8)
Lole et al., 2015	Australia	**NPG** (20) **PG** (16)	**NG**: **M** (9) **F** (11) **PG**: **M** (11) **F** (5)	**NG**: 28.75 (11.19) 18–49 **PG**: 34.8 (16.79) 18–67
Xia et al., 2018	China	**NG (Optimist)** (27) **NG (Pessimist)** (25)	**NG (Optimist)**: **M** (17) **F** (10) **NG (Pessimist)**: **M** (12) **F** (13)	**NG (Optimist)**: 19.67 (1.64) **NG (Pessimist)**: 19 (1.32)
Ulrich & Hewig, 2018	Germany	**NPG** (20) **PG** (20)	**NG**: **M** (18) **F** (2) **PG**: **M** (18) **F** (2)	**NG**: 25.1 (5.78) **PG**: 25.7 (5.72)
Dores et al., 2020	Portugal	**NPG** (23)	**M** (13) **F** (10)	**NG**: 22.5 (3.65) 19–34

*Note* PG = Problem Gambler; NPG = non-problem gambler

M = Male; F = Female

*Study reports two independent experimental trials

When it comes to the ERP components assessed, most studies assessed both the P300 and the FRN components (*k* = 11). Two studies assessed only the FRN, one assessed only the P300, and one assessed the event-related negativity (ERN). For measuring P300 amplitudes, five studies used the mean amplitude, five used the peak amplitude, and two used the peak average. For measuring FRN amplitudes, eight studies used mean amplitude, two used peak-to-peak amplitude, two used peak amplitude, and one used base-to-peak amplitude. The study looking at the ERN reported the mean amplitude Given the positive deflection of the feedback-related response (FRR) to wins, and the negative deflection of the FRR to losses, as well as the significant difference between both outcomes found in two studies (Lole et al., 2013, 2015), the present authors use a different methodological approach to study the FRN, by dividing the FRR into FRN and the FRP (feedback-related positivity).

All studies employed a gambling task comprising: slot machine with a varied number of reels (Alicart et al., 2015; Dores et al., 2020; Luo et al., 2011; Qi et al., 2011; Xia et al., 2018); wheel of fortune (Ulrich & Hewig, 2014, 2018); blackjack (Hewig et al., 2007; Kreussel et al., 2013); an EGM (Lole et al., 2013, 2015); other computerized gambling tasks (Hajcak et al., 2006; Peterburs et al., 2013; Wu & Zhou, 2009; Zhou et al., 2010); and coin toss (Ulrich & Hewig, 2018). Regarding the assessment of the gambling severity, the South Oaks Gambling Screen (SOGS; Lesieur & Blume, 1987) was used in five studies (Dores et al., 2020; Kreussel et al., 2013; Qi et al., 2011; Ulrich & Hewig, 2018; Xia et al., 2018), and the Canadian Problem Gambling Index (CPGI; Ferris & Wynne, 2001) was used in one study (Lole et al., 2013). The Problem Gambling Severity Index (PGSI), a component of the CPGI, was used in one study (Lole et al., 2015). Three studies used the Kurzfragenbogen zur Glücksspielsucht - KFG [Short questionnaire on gambling disorder] (Kreussel et al., 2013; Ulrich & Hewig, 2014, 2018). One study (Ulrich & Hewig, 2018) used the Gambling Related Cognitions Scale (GRCS; Raylu & Oei, 2004) to screen for gambling-related cognitions, the Belief in Good Luck Scale (BIGL; Darke & Freedman, 1997) to assess the feeling of luck, the UPPS Impulsive Behavior Scale (UPPS; Keye et al., 2009) to assess impulsivity, and the Achievement Motives Scale (AMS; Lang & Fries, 2006) to assess motivation for success. To evaluate post-task variables, such as sensitivity and feelings towards reward/punishment, three studies used custom-made structured interviews/questionnaires (Hajcak et al., 2006; Lole et al., 2013; Zhou et al., 2010). Five studies did not use or report the assessment instruments (Alicart et al., 2015; Hewig et al., 2007; Luo et al., 2011; Peterburs et al., 2013; Wu & Zhou, 2009). The methods of each study are presented in Table 2.

**Table 2:** EEG analysis and studies methodologies

Authors	EEG Analysis						Methods		
	ERP Used	Outcomes Assessed	Time Window		Amplitude Measurement	Latency	Instruments	Gambling Tasks	Monetary Reward
Hajcak et al., 2006*	FRN	**Experiment 1**: Big gain Small gain Big loss Small loss. **Experiment 2**: Big gain Small gain Big loss Small loss. Break-even.	**FRN (P)**: 150-350ms **FRN (N)**: (P-350ms)		**FRN**: Base-to-peak amplitude	-----------	Post-task questionnaire	**Experiment 1**: Graphic representation of four doors in a horizontal line to choose one. **Experiment 2**: Choose between one of five doors on each trial.	Course credit, and up to 10$. Could win/lose 5 or 25¢.
Hewig et al., 2007	ERN	Win Loss.	**ERN**: 300-350ms **ERN (Após 3ª e 4ª carta)**: 250-350ms		**ERN**: Mean amplitude	-----------	-----------	Blackjack (Seventeen four)	6€/hour and up to 8.6€.
Wu & Zhou, 2009	FRN P300	Big gain Small gain Big loss Small loss.	**FRN**: 250-350ms **P300**: 250-600ms		**FRN**: Mean amplitude **P300**: Peak amplitude	-----------	-----------	Shown a cue about the monetary reward, and then it is selected a card. Afterwards a feedback stimulus is presented with the valence of reward and magnitude expectancy.	Paid 30 yuans, plus a bonus of about 10.
Zhou et al., 2010	FRN P300	Win Loss.	**FRN**: 200-280ms **P300**: 250-600ms		**FRN**: Mean amplitude **P300**: Mean amplitude	-----------	Post-task questionnaire	Three boxes are shown. After box selection the program showed the third box reward, and the participants were given the choice to switch or stay with the box chosen at the beginning	Start with 30 yuans. Win/lose 25 or go even (0).
Luo et al., 2011	FRN P300	Win Near miss Full miss.	**FRN**: 200-300ms **P300**: 300-550ms		**FRN**: Mean amplitude **P300**: Mean amplitude	-----------	-----------	Slot machine	Start with 30 yuans, then get additional rewards.
Qi et al., 2011	FRN P300	Win Near miss Full miss.	**FRN**: 200-300ms **P300**: 300-600ms		**FRN**: Mean amplitude **P300**: Peak amplitude	-----------	SOGS	Slot Machine	Start with 25 yuans, then get additional rewards.
Kreussel et al., 2013	FRN	Full loss Near loss.	270-320ms **(third card)** 430-480ms **(fourth card)**		**FRN**: Mean amplitude	-----------	SOGS; MINI-DIPS; KFG	Blackjack	6€/hour, plus a reward that could go up to 27.5€.
Lole et al., 2013**	FRN P300	Large wins Small wins Near wins Losses.	-----------		**FRN**: Peak amplitude **P300**: Peak amplitude	**FRN**: 290ms **P3b**: 472ms	CPGI Eye-tracking Subjective experience questionnaire	EGM	6000 credits for a voucher. 7000 would give two.
Peterburs et al., 2013	FRN P300	Clear Win Clear Loss Ambivalent Outcomes.	**FRN**: 230-330ms **P300**: 300-550ms		**FRN**: Mean amplitude **P300**: Mean amplitude	-----------	-----------	Choosing one of two cards	Start with 1000€, then bet 0 or 50€.
Ulrich & Hewig, 2014	FRN P300	Full win Full miss Narrow win Near miss.	**P300**: 300ms-650ms **FRN (P)**: 175ms-275ms **FRN (N)**: 275-450ms		**P300**: Mean peak amplitude **FRN**: Peak-to-peak amplitude	-----------	KFG	Wheel of fortune	10€ or course credit plus 5€/win.
Alicart et al., 2015	P300	Gain Near miss Loss No-information.	340-388ms		**P300**: Peak amplitude	360ms	-----------	Slot machine	Start with 1000 points, then bet 1, 5 or 25.
Lole et al., 2015**	FRN P300 P3b	Large wins Small wins Near wins Losses.	**FRN**: 250-350ms **P300**: 250-650ms **P3b**: 250-600ms		**FRN**: Peak amplitude **P300**: Peak amplitude **P3b**: Peak amplitude	**FRN**: 302ms (PG) 293 (NG) **P300**: 510ms (PG) 506ms (NG) **P3b**: 470ms	PGSI Impulsiveness Questionnaire BIS/BAS scale inventories	EGM	6000 credits for a voucher. 7000 would give two.
Xia et al., 2018	FRN P300	Big win Win Near win Full miss.	**FRN**: 200-300ms **P300**:300-550ms		**FRN**: Mean amplitude **P300**: Mean amplitude	-----------	OPS-C-SF; SOGS; LOT-R; DASS-21	Slot Machine	Start with 500 credits, then get additional rewards.
Ulrich & Hewig, 2018	FRN P300	**Wheel of Fortune**: Full miss Full win Near miss Narrow win. **Coin Toss**: Win Loss.	**Wheel of Fortune** **P300**: 300-650ms **FRN (P)**: 175-275ms **FRN (N)**: 275-450ms	**Coin Toss** **P300**: 250-600ms **FRN (P)**: 150-250ms **FRN (N)**: 200-350ms	**FRN**: Peak-to-peak amplitude **P300**: Mean peak amplitude	-----------	SOGS; KFG; GRCS; BIGL; UPPS; AMS; Structured interview for PG	Wheel of fortune/Coin toss paradigm	Start with 18.5€. 10€/win; plus 4.30€/win on the wheel of fortune, and 4.20€/win in the coin toss.
Dores et al., 2020	FRN P300	Win Loss Near miss after Near miss before.	**FRN**: 150-300ms **P300**: 250-500ms		**FRN**: Mean amplitude **P300**: Mean amplitude	-----------	SOGS; MOCA	Slot Machine	5€/win.

*Note* *Study reports two independent experimental trials

**To assess FRN, studies divided the FRR into FRP for positive outcomes, and FRN for negative outcomes

PGSI = Problem Gambling Severity Index; BIS/BAS = Behavioral Inhibition System/Behavioral Activation System; CPGI = Canadian Problem Gambling Index; SOGS = South Oaks Gambling Screen; KFG = *Kurzfragenbogen zur Glücksspielsucht*; OPS-C-SF = Chinese version of the short form Optimist and Pessimist Scale; LOT-R = Life Orientation Test Revised; DASS-21 = Depression Anxiety and Stress Scale; MINI-DIPS = GRCS = Gambling Related Cognition Scale; BIGL = Belief in Good Luck Scale; AMS = Achievement Motives Scale; MOCA = Montreal Cognitive Assessment

### Findings Regarding P300

For the P300, wins (positive feedback) elicited a larger amplitude than losses (negative feedback) in eleven studies (Alicart et al., 2015; Dores et al., 2020; Lole et al., 2013, 2015; Luo et al., 2011; Peterburs et al., 2013; Qi et al., 2011; Ulrich & Hewig, 2014, 2018; Xia et al., 2018; Zhou et al., 2010). NWs elicited a larger amplitude than losses in eight studies (Alicart et al., 2015; Dores et al., 2020; Lole et al., 2015; Luo et al., 2011; Qi et al., 2011; Ulrich & Hewig, 2014, 2018; Xia et al., 2018). The amplitudes elicited by wins and losses were further modulated by expectancy. In cases of an expected reward, the P300 amplitude for wins was larger than when the reward was unexpected in one study (Wu & Zhou, 2009). When comparing the losses and NWs conditions, three studies showed that NWs elicited a higher P300 amplitude (Alicart et al., 2015; Qi et al., 2011; Xia et al., 2018), two studies showed a larger amplitude for losses (Ulrich & Hewig, 2014, 2018), while three studies showed no differences (Dores et al., 2020; Lole et al., 2013, 2015; Luo et al., 2011). Despite some studies reporting no differences, when deconstructing the NWs before and after the payline, results indicated that NWs before the payline produced a higher P300 amplitude compared to losses, and that losses elicited a higher amplitude than NWs after the payline (Dores et al., 2020). Furthermore, wins led to a higher P300 amplitude than NWs before the payline, and losses led to a higher P300 amplitude than NWs after the payline (Dores et al., 2020).

**Fig. 2:**
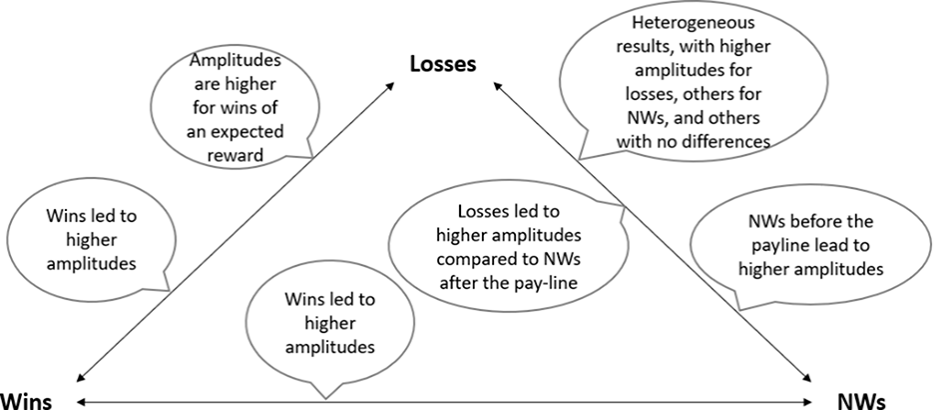
Main findings on P300

For reward magnitude, two studies showed that BWs produced a higher amplitude than SWs (Lole et al., 2015; Xia et al., 2018), while one study showed no difference (Lole et al., 2013). As previously mentioned, one study (Wu & Zhou, 2009) reported that the P300 response to reward magnitude was connected to expectancy, namely when the reward was expected, a BW elicited a higher P300 amplitude. However, when unexpected, there was no difference between a BW and a SW. The P300 was also shown to be dependent on previous outcomes in one study (Ulrich & Hewig, 2018). In a set of three outcomes, depending on when the wins/losses occurred, there could be a different amplitude. For example, getting a win after a win streak (two wins in a row), showed the biggest amplitude. Findings regarding P300 modulation by experimental conditions and outcomes are presented in Fig. 2 and Table 3.

**Table 3:** Reported results

Authors	Results		Limitations
	ERP	Behavioral	
Hajcak et al., 2006*	**Experiment 1**: **FRN**: L > W **FRN**: BW ~ SW **FRN**: BL ~ SL **Experiment 2**: **FRN**: Breaking even ~ L > W **FRN**: BW ~ SW **FRN**: BL ~ SL	Higher level of happiness following wins; Happier for BWs compared to SLs; Happier for SLs compared to BLs.	-----------
Hewig et al., 2007	**ERN**: L > W **ERN**: L > W (high-risk scenario > low risk scenario) **ERN**: high-risk decision > low risk decision	Increased number of points means lower odds of taking another card. Participants with a smaller risk threshold avoided high risk decisions. Participants with higher ERN after a high-risk decision tended to avoid these decisions. Participants with higher ERN after a bad card in medium risk scenarios, tended to more cautious decisions afterwards.	-----------
Wu & Zhou, 2009	**FRN**: Reward magnitude unexpected > reward magnitude expected SW > BW Negative feedback > Positive feedback. **P300**: BW > SW (Expected condition) BW ~ SW (Unexpected condition) Positive feedback > negative feedback (Expected condition > Unexpected condition) Positive feedback > negative feedback (Large magnitude > Small magnitude).	Sensitivity to previous trials; More likely to pick the same card if it led to a previous reward, in comparison to if they lost.	-----------
Zhou et al., 2010	**FRN**: L > W (Action > Inaction) **P300**: W > L (Action ~ Inaction)	Inaction was more prevalent in all the trials; Same choice if the outcome led to good/safe choices; Wins led to more positive responses, which were even higher for action choice.	-------------
Luo et al., 2011	**FRN**: L > NW > W **P300**: W > NW ~ L	The proportion of choosing to bet after: A win (28–60%); A NW (49.7–69.2%); A loss (36.8–58.3%); More rapid decisions after NWs than wins.	-----------
Qi et al., 2011	**FRN**: L ~ NW > W **P300**: W > NW > L	P300 as a predictor of motivational ratings.	Gender was not recorded.
Kreussel et al., 2013	**P300 (270-320ms) NPG**: L > NW (no significant difference between the two for PG) **P300 (430-480ms) PG**: L > NW (for both groups)	**NPG**: Average amount earned was 9.59 €; Less likely to make a risky decision if their ERP showed a significant difference between loss and NW. **PG**: Average amount earned was 6.84 €; Both groups became more cautious in their subsequent gambling behavior following NW.	The sample size was relatively small.
Lole et al., 2013	**FRN**: L > NW; **FRP**: BW > SW **P300**: W > L (BW ~ SW; L ~ NW)	Participants selected the bet low option on 38.9% of trials; BWs were related as more exciting than SWs; SWs were more exciting than losses.	Lack of ecological validity; Rewards capped and restricted to small amounts; Difficult to generalize laboratory findings to actual gambling behaviors. Lack of real financial implications.
Peterburs et al., 2013	**FRN**: bet > no bet; L > W; ambivalent outcomes > W ambivalent outcomes ~ L. **P300**: no bet > bet; W > L > ambivalent outcomes.	-----------	-----------
Ulrich & Hewig, 2014	**FRN**: NW > NAW > L > W **P300**: W > NAW > L > NW	Wins were rated as more positive and motivating compared to losses.	Reduced external validity by making each of the four outcomes equally likely.
Alicart et al., 2015	**P300**: W > NW > L ~ no information**	Five points was the most chosen bet amount, followed by 1 and 25 points; For risky bets the choice depended on the previous outcome. The proportion of high bet choices increased for loss, NW, and no-information trials. After a no-information, tendency was to repeat the same bet.	-----------
Lole et al., 2015	**Amplitude of PG** **FRP**: BW ~ SW **FRN**: L > NW **P300**: BW > SW > NW ~ L **Amplitude of NPG** **FRP**: BW ~ SW **FRN**: L > NW **P300 (P3b)**: BW > SW > NW ~ L	PGs that reported higher scores on the BAS Fun-seeking scale had larger P3b in response to both SWs and BWs, and NWs.	Perceptions of losses as non-reward rather than punishment; Small incentives might elicit smaller responses compared to large incentives.
Xia et al., 2018	**FRN**: L > NW > W > BW **P300**: BW > W > NW > L	A higher bet was more likely to happen after a NW in the optimist group; A higher bet was more likely after a win in the pessimist group.	Small sample size; Laboratory task differs from actual gambling; Participants didn’t use their own money.
Ulrich & Hewig, 2018	**Wheel of Fortune: FRN: PG** (NW ~ L > W ~ NAW); **NPG** (NW ~ L > NAW ~ W); **P300: PG** (W > NAW > L > NW); **NPG** (W > L > NAW > NW). NPG > PG (FRN amplitude); PG ~ NPG (P300 amplitude). **Coin Toss: FRN: WIN**: [**PG** (Lossloss ~ winloss ~ losswin ~ winwin); **NPG** (Losswin ~ winloss ~ winwin ~ lossloss)]; **LOSS**: [**PG** (winwin ~ losswin ~ lossloss ~ winloss); **NPG** (winloss ~ losswin ~ lossloss ~ winwin)]. **P300: WIN**: [**PG** (winwin > losswin ~ lossloss ~ winloss); **NPG** (winwin > winloss ~ lossloss ~ losswin)]; **LOSS**: [**PG** (winwin > losswin ~ lossloss ~ winloss); **NPG** (winwin > losswin ~ lossloss ~ winloss)]. NPG > PG (FRN amplitude); PG > NPG (P300 amplitude).	-----------	Lack of balance between coin toss and wheel of fortune; Certain gambling behaviors are not considered for the control sample; Lack of ecological validity; Small sample size.
Dores et al., 2020	**FRN**: L ~ NW > W; **FRN**: L ~ NW after the payline > W ~ NW before the payline. **P300**: W > L ~ NW; **P300**: W > NW before the payline > L > NW after the payline.	-----------	Small sample size; Lack of ecological validity; No real money involved; Different number of trials.

*Note* W = Win; NW = Near-Win; L = Loss; BW = Big Win; SW = Small Win; BL = Big Loss; SL = Small Loss; NAW = Narrow Win

PG = Problem Gambler; NPG = non-problem gambler

~ = No significant differences

*Study reports two independent experimental trials

**Control outcome, with no presentation of gambling stimuli

### Findings Regarding FRN

All studies showed a larger (more negative) FRN to losses (negative feedback) conditions compared to wins (positive feedback) conditions (Dores et al., 2020; Hajcak et al., 2006; Hewig et al., 2007; Kreussel et al., 2013; Lole et al., 2013, 2015; Luo et al., 2011; Peterburs et al., 2013; Qi et al., 2011; Ulrich & Hewig, 2014, 2018; Wu & Zhou, 2009; Xia et al., 2018; Zhou et al., 2010). This difference in FRN amplitude was shown to be more significant in high-risk scenarios compared to low-risk scenarios in one study (Hewig et al., 2007), and in scenarios where the outcome results from action instead of inaction in one study (Zhou et al., 2010). Additionally, breaking even was shown to elicit the same FRN amplitude as a loss in one study (Hajcak et al., 2006). For NWs, a more negative FRN was shown for this outcome compared to wins in five studies (Dores et al., 2020; Luo et al., 2011; Qi et al., 2011; Ulrich & Hewig, 2014; Xia et al., 2018). Comparing losses to NWs, five studies showed that losses produced a more negative FRN than NWs (Kreussel et al., 2013; Lole et al., 2013, 2015; Luo et al., 2011; Xia et al., 2018). However, one study showed that NWs produced a more negative FRN than losses (Ulrich & Hewig, 2014), while three studies showed no differences (Dores et al., 2020; Qi et al., 2011; Ulrich & Hewig, 2018). Despite these results, one study by dividing the NWs into before and after the payline, shown that NWs after the payline and losses elicited a similar FRN amplitude, and NWs before the payline and wins elicited a similar FRN amplitude, more positive than the FRN elicited by a NW after the payline and losses (Dores et al., 2020).

**Fig. 3:**
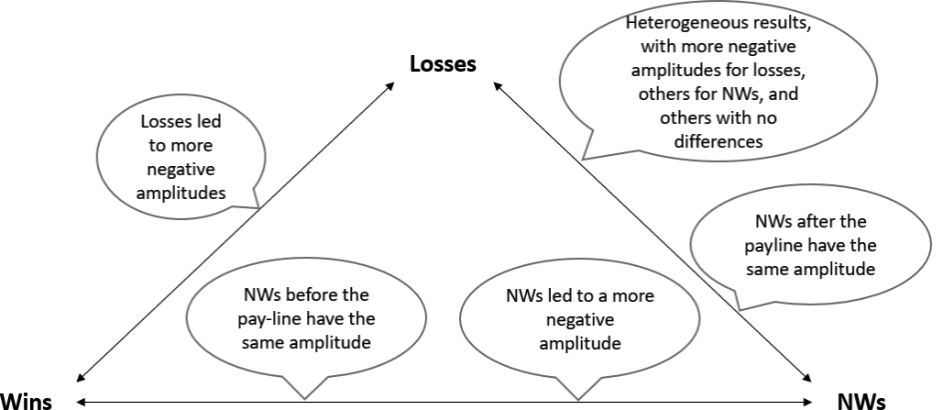
Main findings on FRN

For reward magnitude, three studies reported that SWs produced a more negative FRN than BWs (Lole et al., 2013; Wu & Zhou, 2009; Xia et al., 2018), while two studies showed no differences between them (Hajcak et al., 2006; Lole et al., 2015). Also, FRN did not differentiate between a BL and a SL in one study (Hajcak et al., 2006). The effects of monetary gains on FRN showed a higher FRN amplitude for trials with bets compared to trials with no bets, and a higher FRN for ambivalent outcomes compared to win outcomes (Peterburs et al., 2013). Reward expectancy is also related to FRN activity, and one study (Wu & Zhou, 2009) confirmed this, by showing that FRN was more negative towards an unexpected reward magnitude compared to an expected reward. Additionally, the FRN was shown to be independent of previous outcomes in one study (Ulrich & Hewig, 2018). Findings regarding FRN modulation by experimental conditions and outcomes are presented in Fig. 3 and Table 3.

### Comparison between Problem Gamblers and Non-problem Gamblers

Regarding the differences between PGs and NPGs, three studies compared both groups (Kreussel et al., 2013; Lole et al., 2015; Ulrich & Hewig, 2018). Analyses of the P300 showed that PGs had an attenuated P3b to all outcomes in one study (Lole et al., 2015), and an attenuated FRN to losses in two studies (Lole et al., 2015; Ulrich & Hewig, 2018), implying a hyposensitivity to both wins and losses. However, the results were inconsistent for P300, due to reports of higher P300 amplitudes for PGs in a coin toss task (Ulrich & Hewig, 2018), and a similar P300 amplitude to NPGs in a wheel of fortune task (Ulrich & Hewig, 2018). One of the key differences in FRN amplitude for PGs, was a delay in the differentiation of NWs as more negative than losses, compared to NPGs. For NPGs, this differentiation was in the time window of 270-320ms, whereas for PGs it was at 430-480ms (Kreussel et al., 2013). No other significant differences were noted between PGs and NPGs. Differences between PGs and NPGs are presented in Table 3.

### Behavioral Data

For behavioral data, on average, PGs gained lower monetary reward than NPGs in one study (Kreussel et al., 2013). There was also a tendency to make the same choices if they previously led to a good outcome, showing sensitivity to previous outcomes in two studies (Wu & Zhou, 2009; Zhou et al., 2010). For the outcome rating, in one study, wins were more positive and motivating than losses, and NWs were less emotionally pleasant than losses but were more motivating (Qi et al., 2011).

For betting, risky bets were dependent on the previous outcome. If the participant won a bet before, then the chances of doing it again, or betting at all, were low in two studies (Alicart et al., 2015; Luo et al., 2011). For risky bets, the chance of having another bet got progressively higher from loss to NW, up to no information (Alicart et al., 2015), and for NPGs, they were less likely to perform a risky bet if their ERP data significantly differentiated a NW from a loss (Peterburs et al., 2013). In the case of no information, there was a higher likelihood of repeating the same bet in two studies (Alicart et al., 2015; Luo et al., 2011). Another variable that interfered with the amount bet in one study was the level of optimism or pessimism. Optimists were more likely to place a higher bet after a NW, and pessimists were more likely to have a higher bet after a win (Xia et al., 2018). In one study, the amount won was also reported to be more exciting, when it was a BW, compared to a SW, and in turn, a SW was more exciting than a loss (Lole et al., 2013). In another study, BWs were also associated with a higher degree of reported happiness, and SLs were associated with a higher degree of reported happiness compared to BLs (Hajcak et al., 2006). Also, in one study, for PGs that scored high on the BAS Fun-Seeking Scale (higher desire for new rewards and willingness to participate in rewarding activities (Carver & White, 1994), P3b amplitudes were greater for SWs, BWs, and NMs (Lole et al., 2015). Behavioral data are presented in Table 3.

## Discussion

The complex nature of gambling disorder requires a multilevel approach, combining neurobiological, behavioral, and psychosocial research, for a more comprehensive understanding of a phenomenon that has public health implications. Despite its classification as a mental health condition, there are gaps in understanding the neuropsychophysiological mechanisms underlying excessive and problem gambling behaviors. More specifically, the role of NWs in reinforcing gambling behavior. The present review provided a detailed overview of previous neurophysiological studies in the field, highlighting their characteristics, methodologies, and key findings, specifically regarding two important ERPs - the P300 and FRN. The studies utilized various gambling tasks, including slot machines, blackjack, and computerized tasks, to assess neuronal responses to various gambling outcomes.

Regarding the P300, the studies consistently demonstrated larger amplitudes for wins compared to losses, with additional modulation by expectancy and reward magnitude. The differentiation between NWs and losses yielded inconsistent results across studies, emphasizing the need for further investigation, particularly regarding the position of NWs in the payline of EGMs.

Similarly, the FRN component consistently showed larger (more negative) amplitudes for losses compared to wins across all studies. However, the distinction between NWs and losses was less clear, with some studies reporting conflicting findings. The position of NWs in the payline of EGMs emerged as a significant factor influencing FRN amplitudes, suggesting a nuanced relationship between neuronal responses and gambling outcomes.

Furthermore, comparisons between PGs and NPGs highlighted differences in neuronal sensitivity to gambling outcomes, particularly regarding attenuated responses of the former compared to the latter. Despite some inconsistencies in the literature, PGs consistently exhibited delayed or altered neuronal responses to specific gambling outcomes, highlighting potential markers of gambling severity.

Behavioral data from the studies further complemented the neuronal findings, showing associations between gambling behavior, outcome expectancies, and individual differences in optimism and pessimism. Risk-taking tendencies, betting patterns, and emotional responses to gambling outcomes varied between PGs and NPGs, providing valuable insights into the cognitive and affective processes underlying gambling behavior.

Overall, these findings contribute to elucidating the complex interplay between neuronal responses, gambling outcomes, and individual differences in gambling behavior. By addressing research gaps and inconsistencies, it is possible to set the stage for future investigations aimed at refining theoretical models of gambling behavior and informing prevention and intervention strategies for problem gambling.

Conducting an in-depth evaluation of neurophysiological studies examining near wins (NWs) in gambling using an ERP methodology evidenced several methodological fragilities in the literature that should be addressed. The first of these is the lack of studies comparing PGs and NPGs, because only three of the 15 studies meeting the inclusion criteria compared both groups (Kreussel et al., 2013; Lole et al., 2015; Ulrich & Hewig, 2018). For ERP studies, the results for P300 were reported in a consistent manner and showed its amplitude as a well-defined measure. This is expected because P300 is the most studied ERP (Huang et al., 2015).

However, the FRN was reported in different ways. Some studies reported it based on amplitude value, while others did not. Furthermore, two studies (Lole et al., 2013, 2015), divided the FRN into positive feedback (the deflection is less negative, i.e., closer to zero) and negative feedback (the deflection is more negative, i.e., further away from zero), providing a different analysis on the FRN, that hinders direct comparisons with other studies due to the methodological differences. Addressing these inconsistencies should be a goal for future research to achieve a more consistent and standardized view of studies in this gambling paradigm.

Another area where the literature is lacking is in the use of EGMs as a gambling task. Given the high association between EGMs and problem gambling behavior compared to other types of gambling (Hing & Russell, 2020), more studies need to be undertaken to understand the specific effects of this type of gambling, as well as bringing greater ecological validity to studies. Several studies also highlighted this and other potential limitations such as lack of ecological validity (Dores et al., 2020; Lole et al., 2013, 2015; Ulrich & Hewig, 2014, 2018; Xia et al., 2018), lack of real financial implications (Dores et al., 2020; Lole et al., 2013), and a small sample size (Dores et al., 2020; Kreussel et al., 2013; Lole et al., 2013; Ulrich & Hewig, 2018; Xia et al., 2018).

As expected, for P300, wins elicited larger amplitudes than losses (Alicart et al., 2015; Dores et al., 2020; Lole et al., 2013, 2015; Luo et al., 2011; Peterburs et al., 2013; Qi et al., 2011; Ulrich & Hewig, 2014, 2018; Xia et al., 2018; Zhou et al., 2010). This result is consistent across the literature and is derived from the higher motivational value associated with wins (Polezzi et al., 2010). However, in one study (Qi et al., 2011), this result was partly influenced by an oddball effect, which was shown to result in a higher P300 amplitude compared to wins (Li et al., 2019).

A major point of contention relates to the differentiating of NWs and losses. Three studies reported that NWs elicited larger amplitudes than losses (Alicart et al., 2015; Qi et al., 2011; Xia et al., 2018), whereas two studies reported the opposite (Ulrich & Hewig, 2014, 2018), and four studies reported no differences (Dores et al., 2020; Lole et al., 2013, 2015; Luo et al., 2011). This discrepancy in results highlights the need to account for further variables that might influence P300 amplitude. One of these variables is previous outcomes, that have been shown to influence P300 amplitude (Ulrich & Hewig, 2018), and has been corroborated in the literature (Osinsky et al., 2012). However, while some studies reported higher P300 amplitude for the current outcome after two consecutive wins (Ulrich & Hewig, 2018), other studies showed that the P300 was larger for the current outcome when the previous two had opposite valence (Osinsky et al., 2012). The literature shows that alterations in a string of stimuli, such as an oddball effect, leads to higher P300 amplitude (Jentzsch & Sommer, 2001; Li et al., 2019).

The type of gambling task used should also be considered because the preference for a specific game can lead to a different pattern of brain activity compared to another (Van Holst et al., 2010). For example, EGMs are shown to be more activating than other forms of gambling (Hing & Russell, 2020). The participants´ dysfunctional cognitions are also related to gambling motivation (Mathieu et al., 2018), and should also be considered when interpreting P300 data, especially for individuals that engage in skill games (Myrseth et al., 2010; Toneatto et al., 1997).

Another major factor that is overlooked in most studies is the division of the NW before and after the payline. As the results of Dores et al. (2020) showed, despite the NW producing the same P300 amplitude as a loss, when dividing it into the two outcomes, a NW before the payline was more stimulating than a loss. In turn, a loss was more stimulating than a NW after the payline on EGMs. This is further corroborated by other studies that have shown the motivational aspect of NWs is only seen for NWs before the payline (Clark et al., 2013). The NW before the payline could also reinforce the perception of this outcome as close to a win (Parke & Griffiths, 2004), therefore validating the P300 results.

These results highlight the need to better study the effects of a NW before and after a payline. This is especially important considering that EGMs employ NW scenarios (Barton et al., 2017). The reviewed studies were also inconsistent with regard to reward magnitude. Two studies showed that BWs elicited larger P300 amplitudes than SWs (Lole et al., 2015; Xia et al., 2018), while one showed no differences (Lole et al., 2013). Further studies are needed to examine this differentiation, with further consideration of the previously discussed variables, because some studies reported that P300 amplitudes differ between small and large outcomes, irrespective of valence (Polezzi et al., 2010).

For FRN, losses elicited a more negative amplitude compared to wins in 14 studies (Dores et al., 2020; Hajcak et al., 2006; Hewig et al., 2007; Kreussel et al., 2013; Lole et al., 2013, 2015; Luo et al., 2011; Peterburs et al., 2013; Qi et al., 2011; Ulrich & Hewig, 2014, 2018; Wu & Zhou, 2009; Xia et al., 2018; Zhou et al., 2010), and NWs also induced more negative amplitudes than wins in five studies (Dores et al., 2020; Luo et al., 2011; Qi et al., 2011; Ulrich & Hewig, 2014; Xia et al., 2018). These results were expected because FRN is sensitive to reward valence (Broyd et al., 2012; Kóbor et al., 2015; Walsh & Anderson, 2012). As in the P300 studies, the major point of contention relates to the differentiation of NWs and losses. While five studies reported that losses were associated with a more negative deflection of the FRN (Kreussel et al., 2013; Lole et al., 2013, 2015; Luo et al., 2011; Xia et al., 2018), meaning a bigger expectancy violation in expectancy, one study reported that NWs led to a more negative FRN (Ulrich & Hewig, 2014), and three studies reported no differences (Dores et al., 2020; Qi et al., 2011; Ulrich & Hewig, 2018). Currently, there is no agreed upon reason for this discrepancy in results, and further research is required to understand the effects of NWs on brain potentials.

As with the P300 studies, FRN studies did not generally examine the NWs before and after the payline. Again, the results of Dores et al. (2020) showed that despite the NW producing the same FRN as losses, when dividing it into the two outcomes, a NW before the payline elicited a more positive FRN deflection, similar to a win (compared to a loss), and that a NW after the payline elicited the same FRN amplitude as a loss. The separation of the NW into before and after the payline appears to lead to different results, for both the FRN and the P300. This highlights the need to employ this methodology in future research, due to the results suggesting that NWs outcome could have different results when examining NWs before or after the payline.

Another variable that may explain these discrepancies is risk, due to losses under high-risk scenarios leading to more negative FRN, compared to losses under low-risk scenarios (Hewig et al., 2007). Related to risk, is betting, which leads to more negative FRN compared to scenarios with no bets (Peterburs et al., 2013). Expectancy is another variable to be considered, where unexpected reward magnitudes lead to a more negative FRN (Wu & Zhou, 2009). The effects of expectancy are further corroborated by the literature, which has shown FRN amplitude variation due to expectancy/outcome prediction, being larger to unexpected than expected outcomes, independent of the outcome valence (Alexander & Brown, 2011; Ferdinand et al., 2012; Hauser et al., 2014; Oliveira et al., 2007; Talmi et al., 2013; Walsh & Anderson, 2012).

Moreover, it seems important to consider if the outcome results from a direct action or inaction of the gambler. If the action leads to a loss, the FRN is more negative, compared to a loss that resulted from inaction (Wu & Zhou, 2009). This is expected because negative outcomes resulting from action lead to a more negative response (Kahneman & Tversky, 1982; Zeelenberg et al., 2002), compared to negative outcomes resulting from inaction. However, some results imply that the negative response that comes from action/inaction could be connected to individuals’ tendency to act (Itzkin et al., 2016). Other possible explanations for this discrepancy could be the type of gambling task used or dysfunctional cognitions.

Concerning reward magnitude, the findings were also inconsistent, with three studies reporting that SWs led to a more negative FRN compared to BWs (Lole et al., 2013; Wu & Zhou, 2009; Xia et al., 2018), and two studies reporting no differences (Hajcak et al., 2006; Lole et al., 2015). Further studies are needed to better understand the effects of reward magnitude on FRN activity because some studies reported that the FRN is not sensitive to magnitude (Meadows et al., 2016; Xu et al., 2018), but to valence (Broyd et al., 2012; Kóbor et al., 2015; Walsh & Anderson, 2012). These studies showed there was no difference between two positive outcomes of different magnitudes. The fact that the FRN is modulated by valence is in line with the FRN reflecting a violation in outcome prediction (Alexander & Brown, 2011; Ferdinand et al., 2012; Hauser et al., 2014; Oliveira et al., 2007; Talmi et al., 2013; Walsh & Anderson, 2012), highlighting a negative reward prediction error (Talmi et al., 2013). Therefore, the nature of the FRN may not be suited to assess differential brain responses to different reward magnitudes.

Regarding the differences between PGs and NPGs, given that only three studies compared both groups, there is no substantial body of evidence that differentiates them in terms of EEG activity and ERPs elicited. There was an agreement that the P300 and FRN amplitudes were more attenuated among PGs in two studies (Lole et al., 2015; Ulrich & Hewig, 2018), which is corroborated by attenuated activity in the frontostriatal reward-processing system (Oberg et al., 2011). Regarding the processing outcomes, the major difference was in the time it took to differentiate between NWs and losses. For PGs, in the time window of 270-320ms, there was no differentiation between losses and NWs, while NPGs in the same time window perceived losses as more negative than NWs. Only in the time window of 430-480ms was the difference noticed among PGs (Kreussel et al., 2013). These results might indicate that one of the major differences between PGs and NPGs is a delay in processing outcomes for PGs. Additionally, when identifying the characteristics that differentiate PGs from NPGs, specific aspects should be considered such as higher impulsivity (Kapsomenakis et al., 2018), inflexibility (Marazziti et al., 2008), gambling type, depression, and financial motives (Barrault & Varescon, 2013). A better understanding of these variables is an important step in differentiating brain activity between PGs and NPGs, which ultimately leads to differences between them, such as earned amount (Kreussel et al., 2013).

To further complement EEG data, a few studies also reported several behavioral outcomes. The behavioral data showed that NWs despite being less pleasant are rated as more motivating than losses (Qi et al., 2011). This is supported by data showing activation of reward circuits in the brain that motivates gambling behavior (Clark et al., 2009). Moreover, NWs also appear to influence decision-making (Sundali et al., 2012) and the amount of bet depending on the individual’s characteristics (Xia et al., 2018).

A more comprehensive view of the effects of NWs on gambling behavior is an important step towards the comprehension of gambling decisions, and the influence of this outcome on following decisions, given that individuals that engage with gambling are sensitive to previous trials (Wu & Zhou, 2009; Zhou et al., 2010). Two studies also indicated that the chances of betting after a win were low (Alicart et al., 2015; Luo et al., 2011). Currently, there is a lack of studies that report on the association between gambling outcomes and likelihood of betting. The decreased chances of betting after a win could be related to dysfunctional cognitions reinforced after winning (Monaghan et al., 2009), or it could be related to personal characteristics, such as optimism/pessimism (Xia et al., 2018). For optimists, a higher-risk bet was more likely to be placed after a NW (Xia et al., 2018).

### Limitations of the Review Process

The present review has several limitations that may be addressed by future studies. The first of these is the inclusion of only peer-reviewed empirical studies, which might exclude other outputs that could further enhance the results found. The second limitation is the inclusion of only papers written in English, thus reducing the number of studies found. The third limitation relates to the use of only three databases, possibly excluding papers from other databases. The fourth limitation is not using a meta-analysis. Not considering other ERPs that could potentially explain brain activity elicited by various gambling paradigms could also be a limitation.

## Conclusion

The results of the present review highlight the lack of robust methodologies, a lack of consistent terminology for gambling outcomes, and a lack of studies that compared PGs and NPGs. Given the lack of studies that compare both groups, the results found in the review are insufficient to determine significant differences in electrical brain activity between PGs and NPGs.

Concerning FRN and P300, the results were consistent when differentiating wins (positive outcomes) and losses (negative outcomes) but were inconsistent in differentiating NWs from losses. Given the potential importance of NWs for continued gambling behavior, no consistent results were found on how they affect brain potentials. However, a few studies indicated that NWs can potentially elicit brain ERPs similar to a win, and that this outcome can alter decision-making and the amount of money bet. Furthermore, the findings reported by Dores et al. (2020) emphasize the need to change how studies approach the NW, namely through its division before and after the payline on EGMs.

Studies also overlooked variables that could potentially mediate the association between brain ERPs and gambling outcomes, such as dysfunctional cognitions, preferred game type, or previous outcomes. The role that reward and loss magnitude have on brain activity is also under-researched, with some studies showing that the P300 and FRN amplitudes are influenced by reward magnitude, while others showing no differences, and requiring further research.
